# Analysis of the Cystic Fibrosis Lung Microbiota via Serial Illumina Sequencing of Bacterial 16S rRNA Hypervariable Regions

**DOI:** 10.1371/journal.pone.0045791

**Published:** 2012-10-02

**Authors:** Heather Maughan, Pauline W. Wang, Julio Diaz Caballero, Pauline Fung, Yunchen Gong, Sylva L. Donaldson, Lijie Yuan, Shaf Keshavjee, Yu Zhang, Yvonne C. W. Yau, Valerie J. Waters, D. Elizabeth Tullis, David M. Hwang, David S. Guttman

**Affiliations:** 1 Department of Cell and Systems Biology, University of Toronto, Toronto, Ontario, Canada; 2 Centre for the Analysis of Genome Evolution and Function, University of Toronto, Toronto, Ontario, Canada; 3 Latner Thoracic Surgery Laboratories, University Health Network, University of Toronto, Toronto, Ontario, Canada; 4 Department of Pathology, University Health Network, University of Toronto, Toronto, Ontario, Canada; 5 Research Institute, The Hospital for Sick Children, Toronto, Ontario, Canada; 6 Adult Cystic Fibrosis Clinic, St. Michael's Hospital, Toronto, ON, Canada; Johns Hopkins School of Medicine, United States of America

## Abstract

The characterization of bacterial communities using DNA sequencing has revolutionized our ability to study microbes in nature and discover the ways in which microbial communities affect ecosystem functioning and human health. Here we describe Serial Illumina Sequencing (SI-Seq): a method for deep sequencing of the bacterial 16S rRNA gene using next-generation sequencing technology. SI-Seq serially sequences portions of the V5, V6 and V7 hypervariable regions from barcoded 16S rRNA amplicons using an Illumina short-read genome analyzer. SI-Seq obtains taxonomic resolution similar to 454 pyrosequencing for a fraction of the cost, and can produce hundreds of thousands of reads per sample even with very high multiplexing. We validated SI-Seq using single species and mock community controls, and via a comparison to cystic fibrosis lung microbiota sequenced using 454 FLX Titanium. Our control runs show that SI-Seq has a dynamic range of at least five orders of magnitude, can classify >96% of sequences to the genus level, and performs just as well as 454 and paired-end Illumina methods in estimation of standard microbial ecology diversity measurements. We illustrate the utility of SI-Seq in a pilot sample of central airway secretion samples from cystic fibrosis patients.

## Introduction

Microbes inhabit all environments on earth and contribute to ecosystem function in ways that are only vaguely understood. Decades of culture-based study have provided a solid foundation for our understanding of microbial physiology, genetics, and evolution, but investigations of microbial ecology have been notoriously difficult due to the small size and vast diversity of microbes. Recent developments of culture-independent methods have illuminated the microbial ecosystems that are found in terrestrial and aquatic environments, as well as those intimately associated with organisms. Of particular interest are microbial ecosystems within the human body (the human microbiome), as they are expected to profoundly impact human health and disease. For example, bacterial communities within human intestines are being studied for their effects on atopic and bowel diseases [1], [2], and altered structure of bacterial communities in the vagina is associated with susceptibility to a plethora of diseases including bacterial vaginosis and sexually transmitted infections [3].

The development of next-generation sequencing technology has enabled the large-scale investigation of microbial communities and stimulated broad interest in microbiota research. Similar to earlier studies that relied on Sanger sequencing, most of the next-generation microbiota studies have focused on a portion of the taxonomically informative 16S rRNA gene. 454 pyrosequencing was the first and is still the dominant next-generation sequencing technology applied to microbiota research because it can produce reads of sufficient length for appreciable taxonomic resolution. This need for adequate read length has generally hampered the use of Illumina sequencing by synthesis technologies that produce shorter reads despite the fact that the vastly higher throughput of the Illumina systems would enable much deeper and cost-effective sampling. Several groups have circumvented this problem by overlapping Illumina reads produced by paired-end sequencing (sequencing reads produced from opposite ends of the same template) to provide longer sequence albeit at the cost of decreased taxonomic resolution due to the unavoidable sequencing of the two highly conserved primer-binding regions [4], [5], [6], [7], [8], [9].

Here we propose 16S rRNA gene-based community analysis by Serial Illumina Sequencing (SI-Seq), which provides a taxonomic resolution similar to 454 pyrosequencing with the throughput and cost-effectiveness of Illumina sequencing. SI-Seq is able to get the best of both technologies by adapting Illumina sequencing technology to read three hypervariable regions of the 16S rRNA gene. Here we describe the development of SI-Seq, its validation using single species controls and a mock community, and its comparison to 454 FLX 16S rRNA sequencing of 56 sputum samples collected from Cystic Fibrosis patients.

## Materials and Methods

### Control communities

Genomic DNA was extracted from overnight cultures of five bacterial species (*Bacillus subtilis*, *Pseudomonas aeruginosa*, *Staphylococcus aureus*, *Erwinia amylovora*, and *Burkholderia cepacia*) using the Gentra Puregene Cell kit (Qiagen). Genomic DNAs were quantified using the Qubit Fluorometer (Life Technologies) and mixed in abundances that differed 10-fold.

### CF sample collection and processing

#### Ethics Statement

Protocols for specimen collection, distribution and use were approved by the University Health Network (UHN) and St. Michael's Hospital Research Ethics Boards.

Central airway secretion samples were aspirated intraoperatively using sterile syringes at the time of lung explantation, from consenting patients with an established diagnosis of CF undergoing transplantation in the Toronto Lung Transplant Program between July 2008 and December 2010. Sputum samples were voluntarily produced by consenting CF patients attending the CF clinic at St. Michael's Hospital. Samples were flash frozen in liquid nitrogen and stored in cryovials at −80°C until use. Whole genomic DNA was extracted using the DNeasy Blood & Tissue Kit, with lysozyme pretreatment (Qiagen, Toronto, ON).

### 16S rRNA amplicons

Primers for amplification and sequencing were designed using consensus nucleotides at each position of the Greengenes 16S rRNA alignment while also considering the high melting temperatures (>70°C) required for Illumina technology (Table 1). Primers were assessed for their matching to reference sequences using the “Probe Match” function of the Ribosomal Database Project (http://rdp.cme.msu.edu/probematch/search.jsp). Multiplexing versions of amplification primers V5+791 and V7-1104 had 8-mer barcodes attached to their 5′ ends. Eight barcodes were used for initial SI-Seq development, whereas a subsequent set of 96 barcodes was designed using the barcrawl program [10] for amplification of the CF sputum templates (Table S1).

**Table 1:** Primers.

Primer Name	Primer Sequence (5′-3′)	% Match*
V5+791	GGGKAKCRAACVGGATTAGATACCCBGGTAGTCCWNRCHSTAAACGWTG	70.4
V6+953 (V6a)	AAGCRGHGGADYRTGYGGYTYAATTCGANGMWAMGCGMRRAACCTTACC	67.3
V6-976 (V6b)	CTCACRRCACGAGCTGACGACRRCCATGCASCACCT	88.5
V7-1104	GGSCRTRMKGAYTTGACGTCRYCCCCDCCTTCCTCC	79.7

* RDP Database match (% Bacteria), including perfect match to sequences of ‘Good’ quality and at least 1200nt

Initially each 25ul PCR consisted of 1X Taq buffer (Invitrogen or Fermentas), 1.5 mM MgCl_2_, 0.2 mM each primer, 0.2 mM dNTPs, 50 ng DNA and 0.4 U Taq polymerase (Native, Invitrogen); later reactions were also performed using the Kapa2G Robust Hotstart Ready Mix (Kapa Biosystems), which provided more reliable amplification. Minus-template control reactions were always included. Amplification conditions were: 2 minutes at 94°C; 30 cycles of 30 seconds at 94°C, 30 seconds at 56°C or 58°C, and 30 seconds at 72°C; followed by 5 minutes final extension at 72°C. All reactions were prepared in a sterile PCR hood.

Four independent 25ul PCR reactions were generated for each patient template. Replicate reactions were then pooled and concentrated using purification columns (Fermentas). To ensure removal of primers and any nonspecific amplicons, reactions were then run on 1% low melting temperature agarose gels and amplicons were extracted from agarose using Agarase (Fermentas) or purification columns (Fermentas), according to the manufacturer's instructions. Amplicons were quantified using the Qubit dsDNA BR assay kit (Life Technologies) and equal quantities of product from each patient were pooled before preparation for Illumina sequencing.

### DNA library construction and Illumina sequencing

Libraries were constructed based on Illumina's paired-end library sample preparation protocol with the exception of starting at the ‘A’ base overhang addition, omitting the fragmentation and end repair steps to maintain the 16S rRNA gene amplicons.

Libraries were loaded onto the Illumina cBot cluster station for cluster generation according to the manufacturer's instructions. Sequencing was performed on an Illumina GA-IIx, and the sequencing recipe used is available for download in xml format from the University of Toronto Centre for the Analysis of Genome Evolution & Function (CAGEF) website (www.cagef.utoronto.ca). First strand sequencing began with the Illumina sequencing primer for eight cycles to read the barcode, followed by a denaturation chemistry and hybridization with a mix of the V5 forward and V7 reverse primers at 5 µM final concentration each. After 36 cycles of sequencing, another round of denaturation was performed and a mixture of V6 forward and V6 reverse primers (5 µM each) was hybridized for another 36 cycles of sequencing. The second strand was then synthesized using the paired-end protocol and the same procedures were carried out to generate the barcoded reads and reads for V5, V7 and V6 forward and V6 reverse. Data were pipelined using the standard Illumina computation pipeline. Library construction, sequencing, and data pipelining were performed at CAGEF.

### 454 pyrosequencing

Barcoded amplicons from 56 CF sputum samples were pooled and then split in half for sequencing using SI-Seq (described above) or 454 pyrosequencing; therefore, the same amplicons generated using primers V5+791 and V7-1104 (Table 1) were sequenced by both technologies. For 454 sequencing, amplicons were submitted to The Centre for Applied Genomics (Toronto, ON) for adaptor ligation and one half plate of single read pyrosequencing on a 454 FLX Titanium sequencer. The average raw 454 read length was 386nt (range 40 to 677); QIIME quality trimming (see below) resulted in an average read length of 415nt (range 200 to 573).

### Structuring local version of reference databases

Sequence reads were simulated using JAligner [11] and in-house Java scripts as follows. Sequencing primers (Table 1 or published [4], [5], [12], [13]) were aligned to each taxon in the database (Bergey, Silva, or Greengenes) using JAligner with the EDNAFULL matrix, and gap opening and extension penalties of 15 and 1, respectively. DNA fragments downstream from the aligned primer (SI-Seq), or starting at the 5′ end of the aligned primer (454 and Illumina Paired-End), were then extracted and concatenated. The proportion of each read set that could be assigned to the genus level was determined by classifying the simulated reads using the RDP Classifier [14] (trained with the same read structure) and choosing the taxonomic classification with bootstrap support greater than or equal to 0.7.

### Preparing sequence reads for classification

Sequence data in FASTQ format were subjected to quality control analysis and prepared for taxonomic classification using an in-house Java application made available on CAGEF website (Figure S1). Reads were assigned to patient samples based on the barcode data by concatenating the two barcodes sequences obtained from each of the two individual paired-end reads and comparing the sequences to a reference barcode dataset. Barcode sequences were required to match a reference barcode sequence at 13 of 16 sites to be retained. Read quality was checked using the FASTQ data. Multiple quality-filtering measures were tested with the controls and those with >6 bases with a Phred quality score below 20 were discarded.

Since SI-Seq produces reads in two possible orientations it was necessary to concatenate the two paired-end reads in both orientations (i.e. Read1::Read2 and Read2::Read1) to ensure that one of resulting concatenated sequences was reconstructed in the proper orientation to match the reference database. Paired reads were trimmed to discard the leading 8-bp barcode and then concatenated in both orientations. The concatenated reads were then individually mapped by UBLAST [15] to a Greengenes dataset (reconstructed with simulated reads structured in V5::V6a::V7::V6b orientation) using an identity cutoff of 0.8. The concatenated read that provided the best UBLAST result was retained as the “correct orientation”.

### OTU clustering and taxonomic classification

#### SI-Seq reads

Operational Taxonomic Units (OTUs) were identified using the OTU_PIPE function within USEARCH [15]; this step also identifies and excludes chimeric reads. The appropriate identity cutoff for OTU clustering was determined empirically using the single species controls, as follows. We determined the average pairwise nucleotide identity of SI-Seq structured sequences from all members of the genera *Pseudomonas* (89% average pairwise identity) and *Bacillus* (85%) in the Bergey database. We clustered starting at the average of these two identities (87%) and repeated the process using identities of 97%, 95%, 93%, 90%, 85%, 83%, and 80%. The consensus sequences for each cluster at each identity cutoff were then classified using the RDP Classifier [14] and the clustering identity that most closely recapitulated the single species input with a low level of false positive classification was chosen. Because 87% was found to be the average nucleotide identity within *Pseudomonas* and *Bacillus*, and also had the optimal empirical results with the single species controls, we performed all clustering of SI-Seq data using an identity cutoff of 87%. Seed sequences from each cluster from each community were classified by further clustering with the SI-Seq structured Silva database using USEARCH, with parameters values chosen to ensure the best hit was chosen (--maxaccepts  = 0 and --maxrejects  = 0).

Additional classifications were performed on each read (i.e., not clustered into OTUs) using the RDP Classifier [14]. Reference databases included three versions of the Bergey database: the SI-Seq structured database, the V5 454 simulated dataset, and the full-length database. RDP assignments with greater than 0.8 bootstrap support were incorporated into OTU tables using an in-house PERL script.

#### 454 reads

454 pyrosequencing data files were de-barcoded and quality filtered using the QIIME pipeline [16] with default parameters except for: a minimum quality score of 20, a maximum primer mismatch of 15, and a maximum of 2 errors in the barcode. A separate pipeline was run using a minimum quality score of 25 and a maximum primer mismatch of 5. The resultant reads were analyzed using the QIIME analysis pipeline with default parameters, unless otherwise indicated. Read trimming and filtering were also done separately using Mothur [17] with the following parameters: minlength = 200, maxlength = 300, maxambig = 0, maxhomop = 10, qwindowsize = 25, qwindowaverage = 30, bdiffs = 1, pdiffs = 10.

For both read sets (454 and SI-Seq) questionable OTUs (i.e., below the abundance cutoff) were removed by manual inspection of OTU tables. Abundance cutoffs were determined by run-specific single species controls, as described in the Results section and shown in Tables S2 & S3.

### Community ecology analyses

OTU tables for 454 and SI-Seq sequences were analyzed using QIIME and the R package VEGAN [16], [18]. Rarefaction analyses for assessment of community coverage were performed in QIIME using 100 rarefactions for each sequence sampling step. The alpha diversity metrics Chao1, observed species, and Phylogenetic Diversity (PD) whole tree were estimated in QIIME during rarefaction. The beta diversity metrics Bray-Curtis (BC), unweighted UniFrac (UF), and weighted UniFrac (WUF) were estimated in QIIME on OTU tables rarefied to the lowest number of sequences per sample (454: 478 sequences; SI-Seq: 38,120 sequences).

The reference tree used for estimating phylogenetic based metrics with the SI-Seq data was inferred using full-length Silva database hits. Full length sequences were aligned using gap opening and extension costs of 30 and 10, respectively, and alignments were trimmed to remove gaps at the beginning and end of the alignment that were due differences in sequence length. Phylogenetic inference was performed using the Neighbor-Joining algorithm with 1000 bootstrap pseudoreplicates. Alignment and phylogenetic inference were performed in CLC Genomics Workbench version 4.9 (Århus, Denmark).

Principal components analyses of 454 and SI-Seq data were performed separately in QIIME [16], using BC, UF, or WUF dissimilarity matrices. Procrustes analysis was used to compare the communities in the 454 and SI-Seq datasets in QIIME. Separate analyses were performed on the BC, UF, and WUF derived principal components. 1000 Monte Carlo simulations were performed to assess significance of the Procrustes matrix correlations. Mantel tests with 1000 permutations were also performed in QIIME to assess significance.

Species Accumulation curves were calculated in VEGAN [18], using rarefied OTU tables. Patient samples were randomly drawn and the cumulative number of OTUs was calculated. This was repeated 100 times. An additional curve was calculated after all OTUs with a combined abundance lower than 0.1% were removed from the dataset.

## Results

### Description of SI-Seq method

**Figure 1:**
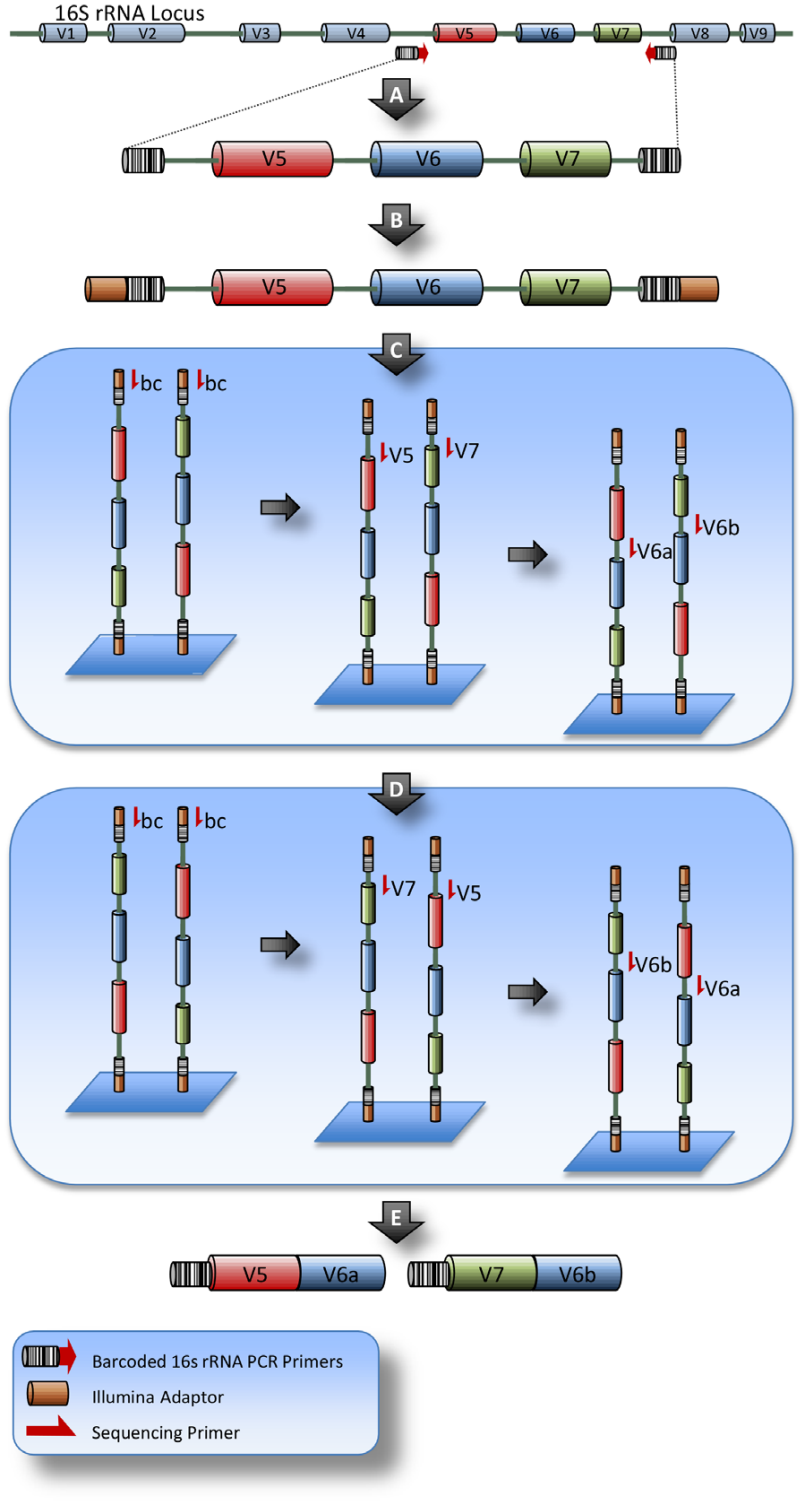
Serial Illumina Sequencing (SI-Seq). (A) Schematic of the 16S rRNA locus with the hypervariable regions indicated as cylinders. The V5 through V7 hypervariable regions are PCR amplified with using universal primers tagged with 8-mer barcodes on their 5′ ends. (B) Ligation of Illumina paired-end adaptors to the 16S rRNA amplicon. (C) 5′ attachment to of each template onto the Illumina flowcell followed by cluster generation (not shown) and serial sequencing of each template using three sequencing primer sets: Illumina sequencing primers to read barcodes (bc); a mix of V5 and V7 primers; and a mix of V6a and V6b primers. Note that each template can bind in either the V5:V6:V7 or the V7:V6:V5 orientation. (D) Fixed templates are flipped on the flowcell and regenerated for pair-end sequencing. Serial sequencing as described in (C) is repeated. (E) SI-Seq data structure showing the barcode, the V5 read and the V6a read, or the barcode, the V7 read and the V6b read.

We sequenced the V5 through V7 region of the 16S rRNA locus to balance taxonomic resolution with fragment size restrictions imposed by Illumina sequencing technology. PCR amplicons of ∼300 bp were generated by amplification of the 16S rRNA gene using bacterial universal primers with 5′ barcodes (Table 1 & Table S1). These amplification products were pooled and libraries were prepared for SI-Seq Illumina sequencing as described in the Materials and Methods. The resulting SI-Seq library consisted of templates with 16S rRNA V5–V7 amplicons flanked by the barcoded universal 16S rRNA PCR primers flanked by the standard Illumina paired-end adaptors (Figure 1). It is important to recognize that each individual SI-Seq template has the potential to bind to the flowcell in either the V5–V7 orientation or the V7–V5 orientation (Figure 1).

As discussed in the Methods section, the SI-Seq library was serially sequenced using multiple template-specific primer sets. The first sequencing step used the standard Illumina sequencing primer to sequence off the Illumina adaptors and into the barcode attached at the 5′ end of the 16S rRNA PCR primers. After eight cycles, this sequencing primer was removed by denaturation and replaced with a mix of V5 and V7 sequencing primers. These primers hybridize in conserved 16S rRNA regions just outside of the appropriate hypervariable regions and sequence into the hypervariable regions. Sequencing was performed with these primers for 36 cycles and followed by another round of denaturation and the addition of two distinct V6 sequencing primers [V6+953 (V6a) and V6-976 (V6b)]. The V6 sequencing primers hybridize to two conserved regions on opposite sides of the V6 hypervariable region and sequence into the V6 region from both orientations. After another round of sequencing, the two V6 primers were removed by denaturation and the clusters were flipped and regenerated using the Illumina pair-end sequencing protocol. After pair-end cluster generation the entire process was repeated by serially sequencing with the Illumina barcode primer, the V5 and V7 primers, and finally the two V6 primers (Figure 1). Overall, a total of six sequencing steps were performed with 144 nucleotides read from the 16S rRNA locus (4 sequencing steps of 36 cycles each) and the eight nucleotide barcode read twice. The primers used and specific number of cycles performed can be easily altered to fit specific needs.

SI-Seq data is generated in two possible orientations depending on how the template binds to the flowcell. The first orientation is barcode:V5:V6a for read one of the pair and barcode:V7:V6b for read two, while the second orientation is the reverse with barcode:V7:V6b for read one and barcode:V5:V6a for read two (Figure 1). These two orientations can be distinguished by a rapid UBLAST analysis [15] as described in the Materials and Methods.

**Figure 2:**
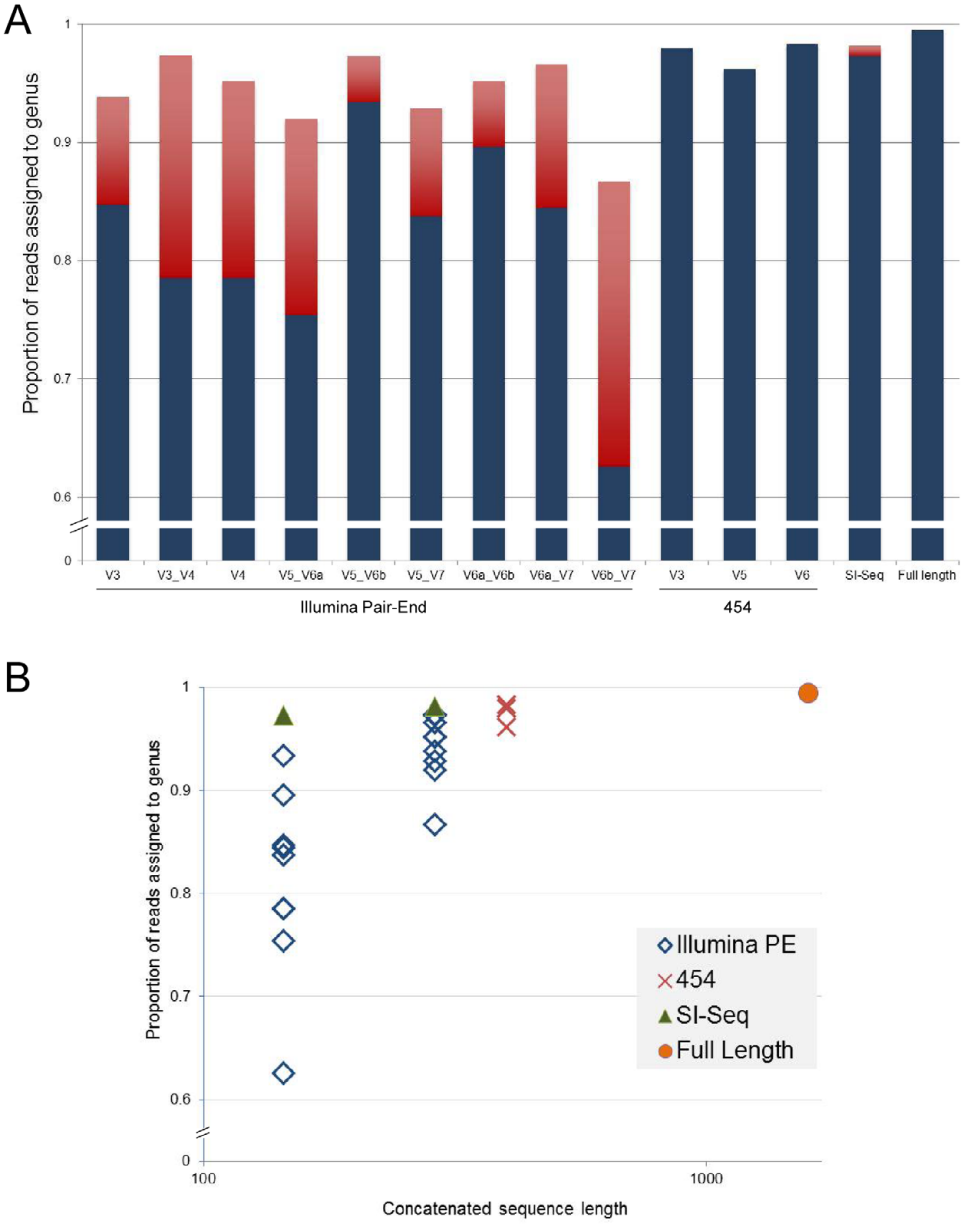
*In silico* simulation of next-generation 16S rRNA sequences based on Bergey database. (A) The proportion of simulated sequences that can be assigned to the genus level using the RDP Classifier for various next-generation 16S rRNA gene sequencing approaches. For the Illumina paired-end data, the blue bars indicate 72nt paired-end reads, while the red bars indicate 144nt paired-end reads (similar to the expected 150 bp Illumina MiSeq reads). For the SI-Seq data, the blue bar indicates 36nt reads, while the red bar indicates 72nt reads. 454 data corresponds to 400nt reads. Full length corresponds to sequencing of the entire 16S rRNA gene. (B) The proportion of reads assigned to the genus level as a function of total concatenated sequence length.

To compare SI-Seq to other commonly used 16S rRNA sequencing protocols (e.g., 454 and Illumina Paired-End), we simulated reads of varying lengths using published primer sequences [4], [5], [12], [13]. From these simulated reads we calculated the proportion of reads that could be assigned to the genus level using the RDP Classifier [14]. As shown in Figure 2, SI-Seq and 454 were similar in their ability to produce a high proportion of reads that could be assigned to the genus level. These data indicate that SI-Seq is in theory comparable to 454 in its ability to produce reads with enough sequence variation for good taxonomic assignment, yet being Illumina-based, produces over 100-fold more reads at similar cost.

### Validation with single species controls

Amplicons from either *Bacillus subtilis* or *Pseudomonas aeruginosa* genomic DNA were sequenced separately by SI-Seq and the resulting reads were used to determine the overall quality of the SI-Seq data and how quality filtering influenced the accuracy of taxonomic classification.

Filtering the sequences to obtain the optimal dataset is a balance between retaining too many sequences of poor quality that may give erroneous taxonomic classifications in order to favor dataset size (type I error) versus discarding too many moderate quality sequences in order to favor dataset quality (type II error). We filtered sequences for quality using four different cutoffs and then classified the retained sequences using the RDP Classifier [14] (Table S2); RDP reference sequences were trimmed to retain only those regions of the 16S rRNA locus recovered by the SI-Seq protocol.

**Figure 3:**
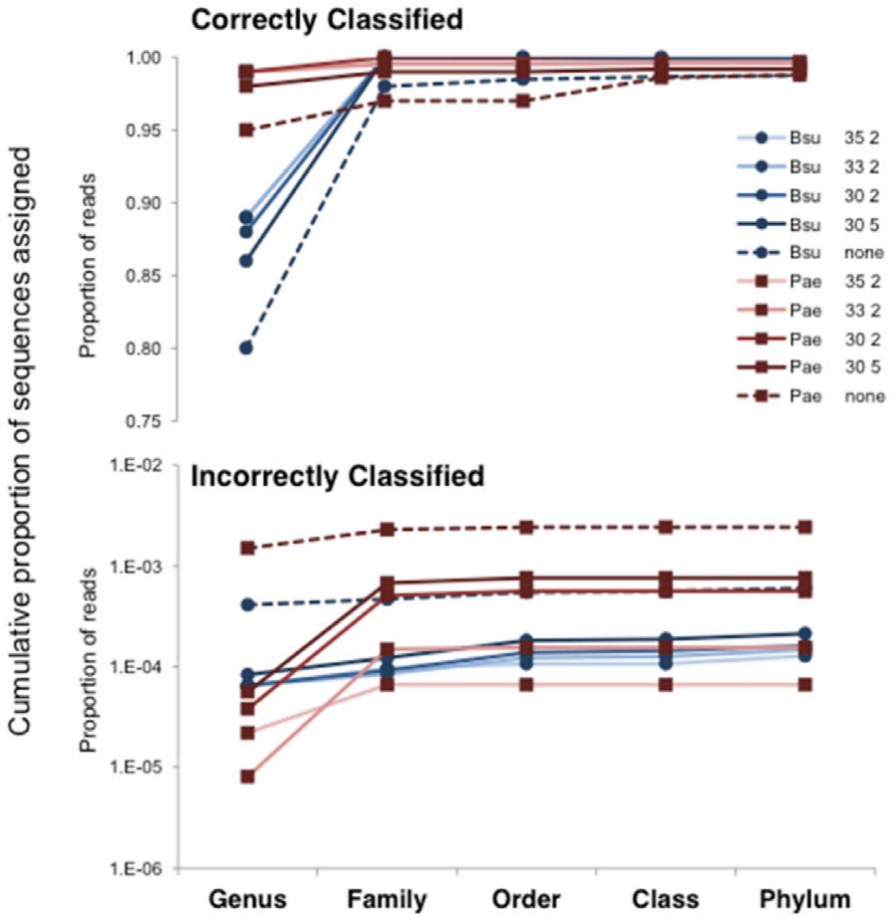
The effect of quality filtering on taxonomic classification. Cumulative distributions are shown for the proportion of sequences assigned to a particular level of taxonomy. Correctly classified sequences are shown in the top panel; incorrectly classified sequences are shown in the bottom panel. Bsu  =  *B. subtilis*; Pae  =  *P. aeruginosa*. The numbers following Bsu and Pae indicate the quality filtering applied. The first number is the Phred score cutoff and the second number is the number of sites allowed with a quality score below the Phred cutoff. For example, 35 2 indicates that sequences were only retained if they had two or fewer sites with a Phred score lower than 35. “none” indicates no quality filtering was performed.

We calculated the rate of taxonomic classification error by calculating the number of SI-Seq sequences whose taxonomy was inconsistent with *B. subtilis* or *P. aeruginosa*. Across several SI-Seq development runs, this average rate of classification error was ∼0.03%. Figure 3 shows the cumulative distributions of sequence proportions assigned to each taxonomic level, for both the correct and incorrect classifications. These data highlight the usefulness of quality filtering: the stricter the quality filter, the larger the proportion of sequences assigned to the genus level. This was particularly apparent for *B. subtilis*. Also evident is that the overwhelming majority of the single species datasets are comprised of sequences that are classified correctly.

Because every Illumina run varies in quality, we included a single species control as one barcoded sample to empirically determine the rate of classification error. This value is then used as a lower, run-specific threshold for elimination of low abundance OTUs.

### Validation with a mock community

**Figure 4:**
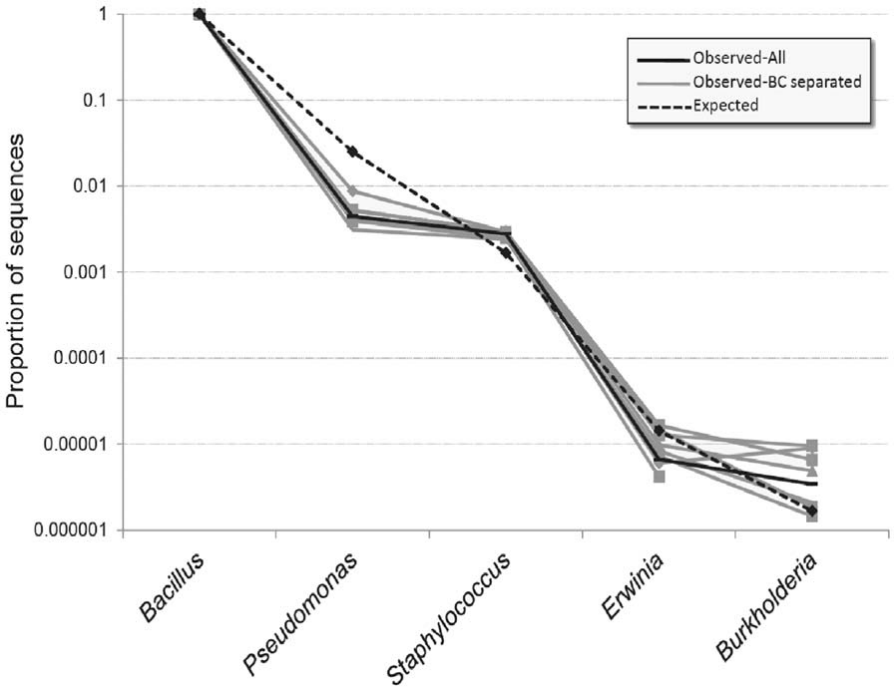
Observed and expected abundances of taxa in mock community control. Observed abundances are given as totals and as barcode-separated datasets. Expected abundances have been corrected for the number of rRNA operons present in each species' genome.

A mock community control was sequenced to determine whether PCR amplification introduced taxonomic biases and to identify the dynamic range of SI-Seq taxon detection. Our control community consisted of genomic DNA from *Bacillus subtilis*, *Pseudomonas aeruginosa*, *Staphylococcus aureus*, *Erwinia amylovora*, and *Burkholderia cepacia* that were mixed at abundances that differed from each other by an order of magnitude. As shown in Figure 4, the observed taxon abundances were nearly identical to those expected. Furthermore, we were able to detect taxon abundances that ranged over five orders of magnitude. The taxon of lowest abundance, *B. cepacia*, had only 120 pg of total genomic DNA added to the original pool, of which less than 1,000^th^ of which would be 16S rRNA; thus, the reliable detection of *Burkholderia* in all separate barcoded samples verifies the repeatability and sensitivity of SI-Seq.

### Comparison of SI-Seq to 454 FLX Titanium

**Figure 5:**
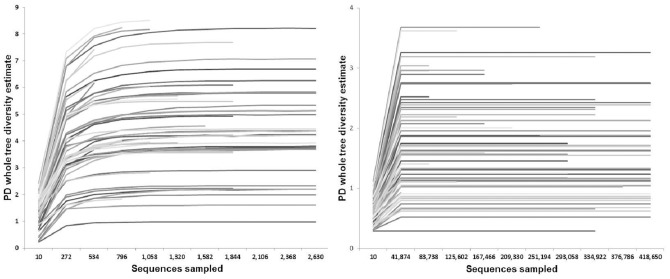
Rarefaction curves from 100 resamplings of each patient's community at different sequencing depths. The y-axis indicates the average Phylogenetic Diversity (PD) whole tree diversity estimate for each sample at each sequencing depth. Data shown are from the 454 OTU table (left), and SI-Seq OTU table (right). Each line corresponds to one sample listed in Table 3.

To further validate SI-Seq, we sequenced 56 sputum samples collected from CF patients using both SI-Seq and 454 FLX Titanium. Samples for both platforms were taken from the same PCR stock to ensure no PCR bias. Sequence reads from 454 and SI-Seq were filtered for quality and de-barcoded either using QIIME (454) or a pipeline developed in-house (SI-Seq) (as described in Materials and Methods). The range of sequences obtained per sample differed substantially between the two sequencing approaches: between 478 and 11,405 quality reads were obtained using 454, whereas between 38,120 and 859,764 quality reads were obtained using SI-Seq (Table 2). The average length for the 454 reads was 415nt, with 33% of the reads shorter than the average. A rarefaction sampling analysis indicated that the number of reads obtained for each patient sample saturated the community diversity in all cases for the SI-Seq data and in most cases for the 454 data (Figure 5). Both rarefaction analyses suggest that ∼1,000 sequences would be sufficient for characterizing the majority of bacteria present in these CF lung communities. The saturation of community diversity with such low read numbers indicates that a much higher degree of multiplexing would further reduce per patient sequencing costs without reducing community information.

**Table 2:** Cystic Fibrosis samples.

		Number of Sequences					
	Sample	Time 1		Time 2		Time 3	
Patient	type	SI-Seq	454	SI-Seq	454	SI-Seq	454
0002	Sputum	319,749	11,405	339,814	7,842		
0004	Sputum	474,226	8,467	443,729	3,941		
0005	Sputum	547,415	7,973	472,506	3,703		
0006	Sputum	859,784	8,008	824,181	8,471		
0007	Sputum	484,648	4,299	538,229	6,587	613,251	5,527
0028	Sputum	383,201	1,650	347,991	3,179		
0035	Sputum	488,906	1,620	363,620	2,008		
0037	Sputum	286,778	2,069	205,463	581		
0052	Sputum	86,380	570	103,260	1,876	100,212	1,089
0060	Sputum	567,023	1,877	799,027	4,760	438,495	4,721
0064	Sputum	418,650	4,075	241,436	4,162	495,277	4,138
0068	Sputum	437,179	3,189	328,141	3,248		
0073	Sputum	360,834	5,418	468,731	6,364		
0077	Sputum	517,309	6,827	576,069	5,161	488,426	6,848
0082	Sputum	554,580	7,614	692,440	8,516		
0083	Sputum	315,906	5,127	509,827	6,394	139,260	580
0089	Sputum	288,846	938	167,530	1,230		
0092	Sputum	38,120	478	81,624	526	315,702	2,088
0096	Sputum	378,450	960	371,370	1,400	299,837	1,489
0104	Sputum	349,007	1,855	157,340	1,276		
0105	Sputum	46,065	1,170	159,400	980		
0106	Sputum	354,331	1,999	190,479	683		
0107	Sputum	183,695	947	221,825	1,392		
0109	Sputum	77,311	1,176	97,820	1,892		
S1	Airway	11,426,004					
S2	Airway	7,037,404					
S3	Airway	1,385,423					
S4	Airway	1,448,413					
S5	Airway	7,191,912					
S6	Airway	2,149,307					
S7	Airway	3,271,206					
S8	Airway	3,380,628					
S9	Airway	6,546,059					
S10	Airway	2,495,002					

**Figure 6:**
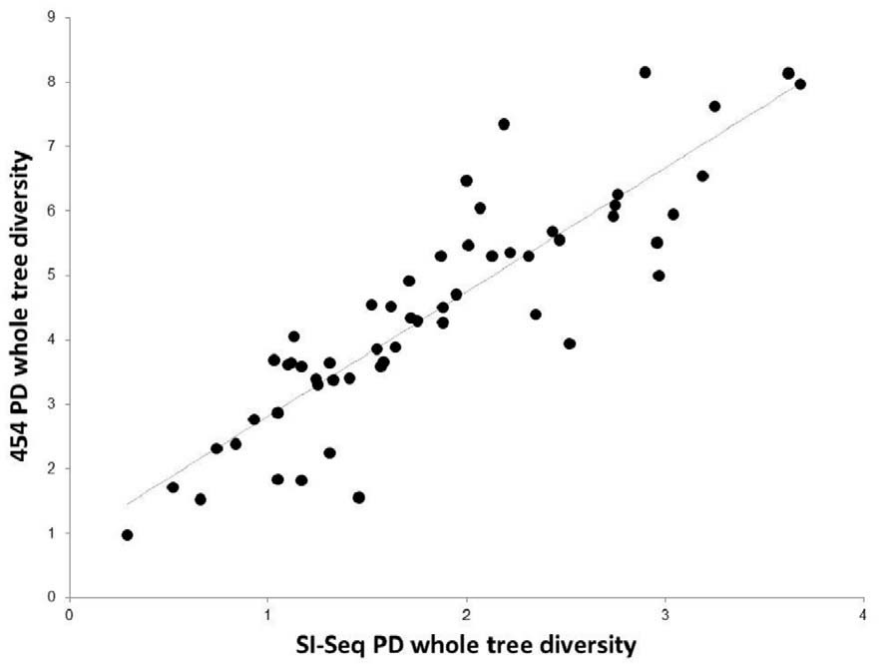
Comparison of phylogenetic diversity (PD). Whole tree PD diversity measurements from the 454 and SI-Seq analysis using rarefied OTU tables with 478 reads per patient for 454 and 38,120 reads per patient for SI-Seq. Very strong correlation is observed between the two approaches.

To compare within sample diversity estimates from 454 and SI-Seq communities, we calculated alpha diversity (Phylogenetic Diversity) on rarefied OTU tables (454: 478 reads per patient; SI-Seq: 38,120 reads per patient). It is important to use rarefied data to normalize the number of sequences per sample; this is because samples with greater sequencing depth are expected to have higher diversity due to sequence coverage alone. Alpha diversity metrics from 454 and SI-Seq data were significantly correlated (Spearman's rho: 0.90; P<2.2×10^−16^; Figure 6).

We used the RDP Classifier to determine the number of reads from each approach that could be classified down to the genus level. Quite strikingly, an average of only 56% of 454 reads were classified to the genus level, while 95% of SI-Seq reads carried this resolution (Table 3). Trimming the 454 reads to include only those regions sequenced by SI-Seq increased this percentage to 61%. Separate quality filtering and processing of raw reads using Mothur [17] increased the percentage of reads assigned at the genus level to 85%. These results support previously published findings showing that the 454 platform has a substantially higher error rate than the Illumina platform, particularly when generating only very short Illumina reads as is done in the SI-Seq protocol [19], [20], [21].

**Table 3:** Taxonomic Classification Comparison.

			Proportion of Sequences Classified to Each Taxonomic Level					
Platform	Template^1^	Ref.DB^2^	Domain	Phylum	Class	Order	Family	Genus
454	PAO1	FL	0	0	0.29	0	0.55	0.16
454	PAO1*	FL	0	0	0.17	0	0.20	0.63
454	PAO1	V5	0	0	0.25	0	0.58	0.17
454	PAO1*	V5	0	0	0.14	0	0.20	0.65
454	PAO1	SI-Seq	0.76	0.18	0.06	0	0	0
SI-Seq	PAO1	FL	0.91	0.09	0	0	0	0
SI-Seq	PAO1	V5	0.56	0.37	0.07	0	0	0
SI-Seq	PAO1	SI-Seq	0	0	0.03	0	0.06	0.91
454	CFS	FL	0.01	0	0.14	0.02	0.27	0.56
454	CFS*	FL	0.01	0	0.06	0	0.08	0.85
454	CFS	V5	0.01	0	0.13	0.02	0.28	0.57
454	CFS*	V5	0.01	0	0.05	0	0.08	0.86
454	CFS	SI-Seq	0.82	0.13	0.04	0	0	0.01
454	CFS-SS	SI-Seq	0.14	0.05	0.10	0.04	0.06	0.61
454	CFS-HQ	V5	0.01	0	0.13	0.02	0.28	0.56
SI-Seq	CFS	FL	0.91	0.06	0.01	0.01	0	0.01
SI-Seq	CFS	V5	0.62	0.27	0.07	0.02	0	0.01
SI-Seq	CFS	SI-Seq	0.01	0	0.01	0	0.02	0.95

1 Template sequenced: PAO1, *P. aeruginosa* PAO1; CFS, CF sputum community; CFS-SS, CF sputum community with 454 reads trimmed to correspond to the SI-Seq data format; CFS-HQ, CF sputum community using only high quality 454 reads (Q>25). *indicates reads were trimmed using Mothur [17] instead of QIIME [16].

2 Reference Database: FL, full length database sequences; V5, all database sequences trimmed to 400 bp starting at our V5 primer; SI-Seq, database sequences formatted to correspond to the SI-Seq read structure.

To determine whether the high proportion of genus level assignments for the SI-Seq data were the result of incorrect high-confidence classifications due to the short SI-Seq read length, we compared the classifications of the single species control data generated by 454 and SI-Seq runs (Table 3). We also used multiple versions of the reference database to ensure that the database structure did not bias the results, including full length reference sequences, reference sequences trimmed to 400 bp starting at our V5 primer, and SI-Seq formatted reference sequences. When classifying each dataset with the appropriately formatted reference database, this analysis remarkably showed that 91% of reads from the single species control (*P. aeruginosa* PAO1) were correctly classified with SI-Seq data compared to only 16% of the 454 data.

**Figure 7:**
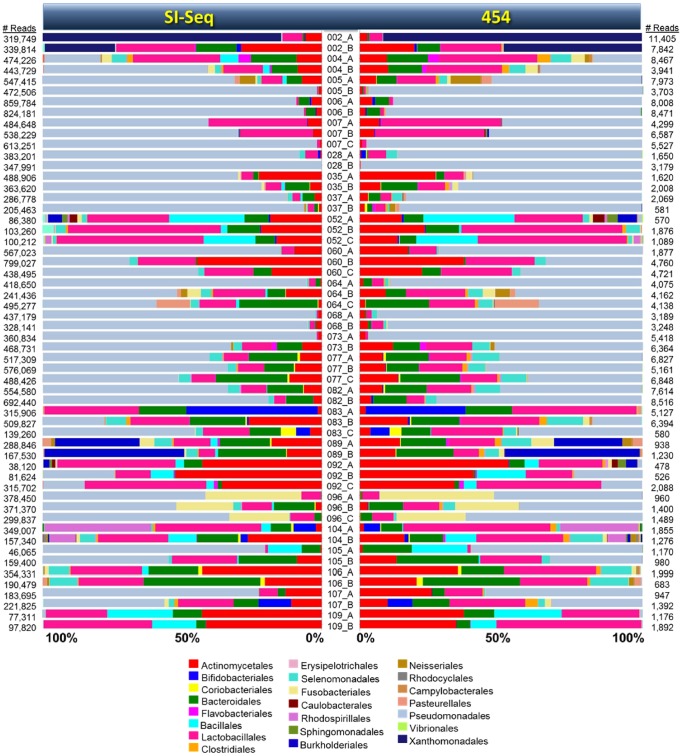
Relative proportions of bacterial orders found in each sample as assessed by SI-Seq and 454 sequencing approaches. Note that the relative taxonomic structure of the communities inferred for each sample are nearly identical using the two methods, although SI-Seq generates ∼100-times more data, thereby permitting a higher level of resolution and larger dynamic range. Data is based on OTUs collapsed to the order level using the MEGAN ver4 software package [35]. Sample IDs are presented in the center of the figure. Relative abundance of bacterial orders for each sample are presented in mirror order for SI-Seq on the left and 454 on the right. The color code for each bacterial order is presented on the bottom. The number of reads (sequences) for each sample are presented on either side.

We compared the 454 and SI-Seq community profiles visually and statistically to verify their similarities. We first compared the per-sample distribution of bacterial orders obtained from the 454 and SI-Seq OTU tables (Figure 7). Despite differing sequencing methods, OTU clustering thresholds, and taxonomy classification approaches, the composition of each community for each sample was remarkably similar between the 454 and SI-Seq datasets. We next tested for statistical significance using a Procrustes analyses to determine whether the OTU dissimilarity matrices obtained from each approach were statistically indistinguishable. We applied a variety of distance metrics and found that the match between community profiles generated by 454 and SI-Seq were highly significant regardless of the dissimilarity matrix used (Table 4; Figure S2).

**Table 4:** Procrustes comparisons of 454 and SI-Seq PCA plots.

Dissimilarity matrix used for PCA plot	M^2^ †	P value*
Bray-Curtis	0.257	<0.001
Unweighted UniFrac	0.649	<0.001
Weighted UniFrac	0.325	<0.001

† Goodness of fit between 454 and SI-Seq PCA plots

* Based on 1000 random permutations of SI-Seq matrix

### Application of SI-Seq to study the ecology of the CF lung

To demonstrate the utility of SI-Seq, we performed additional analyses on the CF sputum samples to begin our investigation of CF lung ecology, although a more in depth analysis of this subject will be completed in the future with a larger patient cohort.

**Figure 8:**
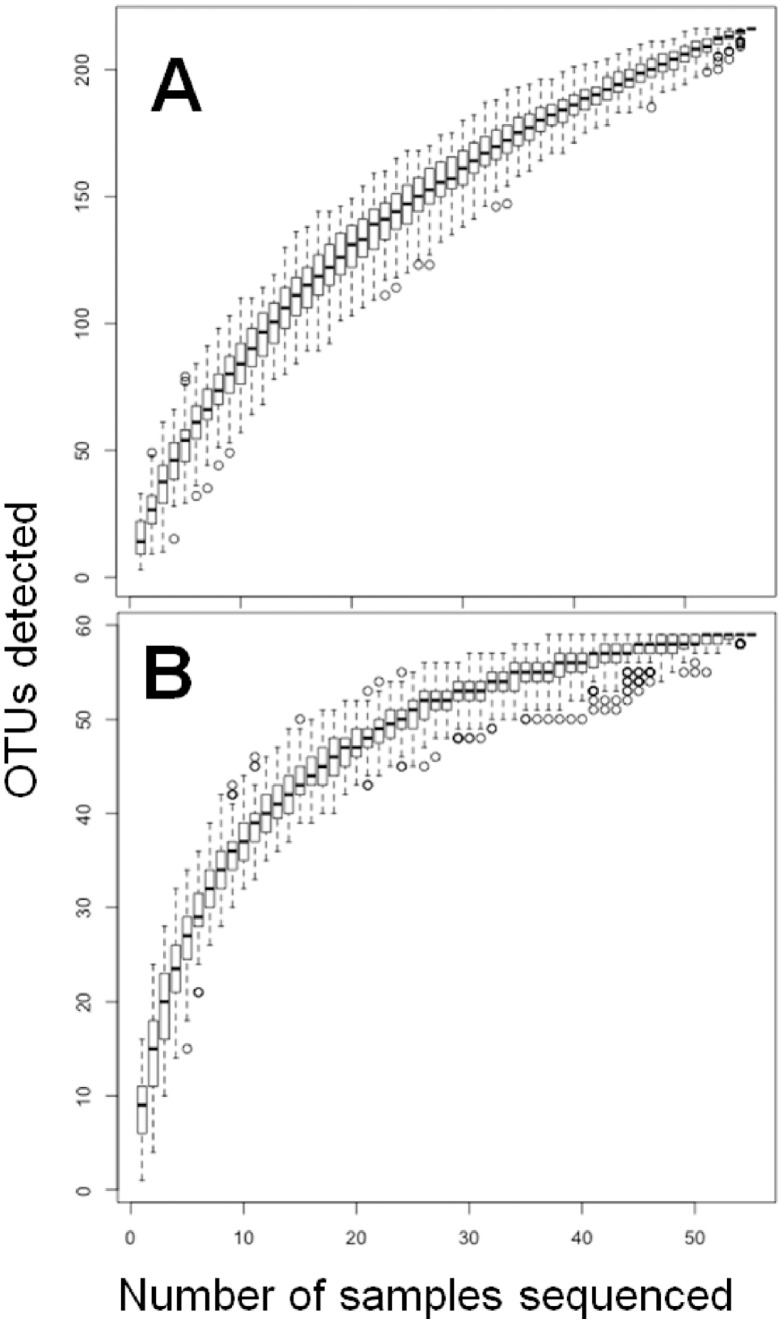
Species Accumulation (SA) analysis. SA plots showing the increase in OTUs detected with the addition of each patient sample. Each bar represents 100 random draws (without replacement) of samples from the sample pool. Panel (A) shows the curve obtained using all of the OTU data whereas panel (B) includes only OTUs with abundances greater than 0.1%.

We used a Species Accumulation curve to determine if the bacterial diversity observed in our patient cohort represented the overall bacterial diversity present in CF lungs. This approach measures how many new OTUs are identified as additional samples are cumulatively added to the analysis. As shown in Figure 8A, the number of OTUs increased quickly between 0 and 20 samples, and began to plateau by the end of our sampling; this indicated that we have largely saturated the diversity found in this environment. Many of the OTUs detected with increased sputum sampling were rare, as reduction of the dataset to include only those OTUs with a total abundance greater than 0.001 resulted in a plateau around 25 sputum samples (Figure 8B).

**Figure 9:**
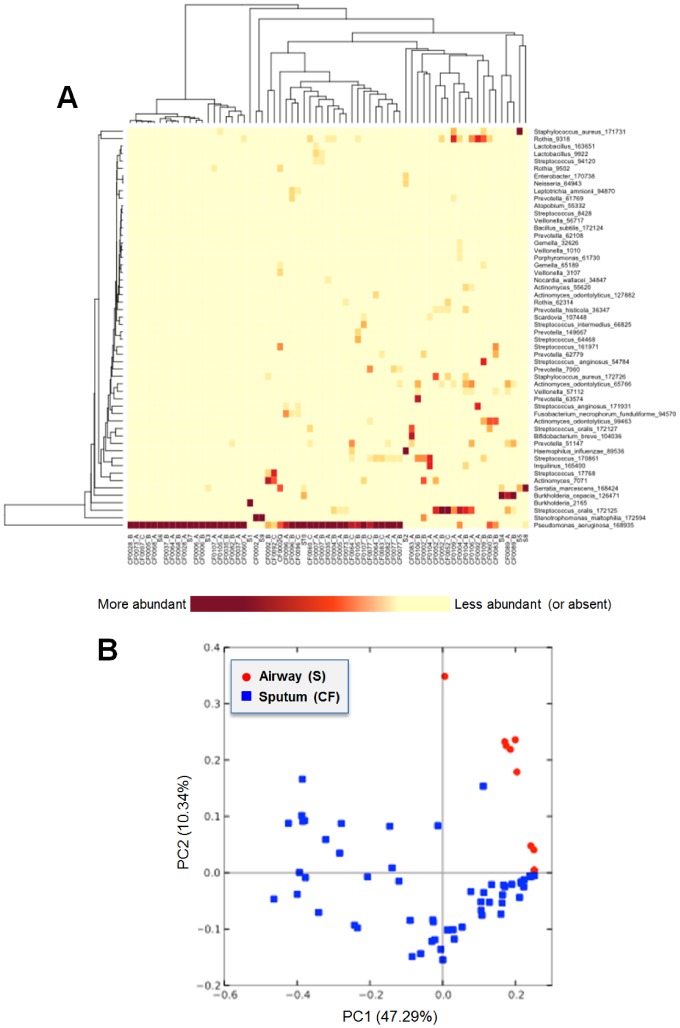
Comparison of bacterial communities in CF sputum and lung airways. (A) Heatmap showing samples from sputum (CF prefix) and lung airways (S prefix) often cluster together. Sample suffixes ‘A’, ‘B’, and ‘C’ indicate the time point for longitudinal samples. For better visualization, OTUs were only included if they had a total abundance greater than 10%. (B) Principal coordinates analysis plot highlighting the sputum (blue squares) and lung airway (red circles) samples. This plot was obtained using the weighted UniFrac estimates for community dissimilarity.

We also performed SI-Seq on ten samples extracted from mucus plugs obstructing the airways of lungs surgically removed from CF lung transplant patients, and combined these results with those from the 56 sputa discussed above. Clustering of community profiles from these two sample types indicated substantial overlap in community composition of sputum and lung airway samples (Figure 9A). The heatmap in Figure 9A shows three types of patient samples: (1) those with only *P. aeruginosa*; (2) those with predominantly *P. aeruginosa* with additional minor constituents; and (3) those with little or no *P. aeruginosa*, but either a diversity of other bacterial species or a community dominated by *Streptococcus* or *Burkholderia*. The ten airway samples were evenly divided among these three groups. Despite this similarity, a principal components analysis using weighted UniFrac dissimilarities showed a separation of the sputum and lung airway samples (Figure 9B). This suggests that when phylogenetic distance is taken into account (as was done with the weighted UniFrac but not the Euclidean distance based heatmap), sputum and airway samples slightly differ in their microbiota.

We also compared our SI-Seq data with clinical culture data and found complete agreement (data not shown). There were no samples in which a particular bacterial species was cultured but not detected using SI-Seq.

## Discussion

Targeted sequencing of taxonomically informative regions of the 16S rRNA gene has been instrumental in describing and comparing microbial communities and ecosystems. Associating changes in these microbiota with disease etiology or ecosystem function requires a method that fulfills the following criteria: good taxonomic resolution; deep enough sequencing to ensure community saturation; and a cost that permits multiple biological replicates and fine temporal or spatial sampling. Our side-by-side comparison of 454 and SI-Seq profiling of single species controls and CF sputum samples revealed the superiority of SI-Seq regarding each of these criteria; below we discuss these in turn.

SI-Seq profiling permits as good or better taxonomic resolution as 454 profiling. Analysis of CF communities using the RDP Classifier found that a maximum of 85% of the 454 reads were classified to the genus level, compared to 95% of SI-Seq reads. This result seems counter-intuitive since the 454 reads are substantially longer than SI-Seq reads. However, highly conserved regions are included in the 454 reads and these regions do not segregate for sufficient or appropriate genetic variation to discriminate genera. SI-Seq passes over these highly conserved regions and reads only hypervariable regions that permit greater taxonomic resolution.

An additional factor affecting taxonomic resolution is the error profiles of the two platforms. It is well established that the 454 platform has an approximately 10-fold higher error rate compared to the Illumina platform [19], [20], and most Illumina sequencing errors are generated in later cycles [21]. Because SI-Seq uses only very short reads, it generates data with an extremely low error rate. This conclusion is supported by our classifications of CF microbiome reads trimmed to match the SI-Seq format, which allowed direct comparisons of 454 and SI-Seq on real samples using the same data format and the same reference database; this trimming only increased the percent of reads assigned to genus by 5% (from 56% to 61%). The substantial difference that remains between 454 and SI-Seq (61% vs. 95% of reads classified to the genus level, respectively) can only be explained by sequencing error (Table 3).

Our analyses of both control and real samples show that bacterial communities profiled using the two approaches were significantly correlated, even though different parameters were used for OTU clustering, different reference databases were used for taxonomic classification, and different reference trees were used for phylogeny-based diversity metrics. The convergence of these sequencing methods and analysis pipelines on a similar picture of the CF lung community speaks to SI-Seq being a robust method.

In addition to superior taxonomic resolution, SI-Seq provides a major cost advantage relative to 454 by producing over 100-fold more data per dollar. Clearly though, it is a unique and more complicated protocol, which requires the careful addition of multiple sequencing primers during the sequencing run and additional data processing steps to deal with the structured reads. Nevertheless, the incredibly high throughput of the Illumina platform provides a compelling case for using SI-Seq. Assuming that the Illumina GA-IIx and MiSeq platforms can sequence 40M and 15M templates per channel per run, respectively; SI-Seq should produce over 400K or 150K reads per sample when using 96-fold multiplexing per channel. Importantly, pooling of SI-Seq samples is performed after PCR, but prior to sample preparation, cluster generation and sequencing; therefore, only one pooled-sample needs to be carried through the sample preparation protocol for all of the sequencing. The net result is that the cost of performing high-multiplex SI-Seq is in the range of tens of dollars per sample, while still obtaining as many as 400K sequences per sample. Multiplexing can be increased for simpler communities (such as the CF lung) if lower depth coverage is needed, or if amplicons are run on machines with higher throughput, such as the Illumina HiSeq.

Paired-end Illumina sequencing of the 16S rRNA gene has been proposed by a number of authors [4], [5], [6], [7], [8], [9], particularly since the Illumina MiSeq can currently produce read lengths of 150nt, and are expected to reach 250nt within the next couple of years. While these approaches have great merit, they have some of the same issues as 454, namely: they are forced to sequence through conserved regions of the 16S rRNA gene; they have a higher error rate since the reads are longer and error rates increase dramatically near the end of long Illumina reads [21] (with the caveat that some Illumina-based methods overlap the ends of the two paired-reads to reduce the overall error); and they cost more per reaction and take longer to run due to the fixed cost and time accrued per Illumina sequencing cycle. SI-Seq, as described here, runs in 144 total cycles, while Illumina-based microbiome approaches need at least 144nt paired-end runs (288 total cycles) to get taxonomic resolution similar to 454 and SI-Seq (Figure 2). So while paired-end Illumina approaches are initially simpler to apply than SI-Seq, they cost at least twice as much per sample and take twice as long to run.

While this study focuses on the interrogation of the V5, V6 and V7 hypervariable regions of the 16S rRNA locus, it should be clear that any region or locus can be analyzed by SI-Seq if appropriate PCR and sequencing primers can be designed. Additionally, the methods can be easily adapted to sequence even larger numbers of regions or provide longer reads for each region. In general, SI-Seq has many potential applications, including targeted re-sequencing of amplicons for SNP detection and multilocus sequence analysis.

Our study of the CF lung microbiota indicated that SI-Seq produces results consistent with laboratory culture and the published literature [22], [23], [24], [25], [26], [27], [28], [29], [30], [31], [32], [33]. This very preliminary analysis reveals some distinction between samples collected from the CF sputum and those collected from airway mucus plugs from explanted lungs. Sampling the microbiota present in mucus plugs is important for determining whether some taxa are truly present in the lower airways, or present in the more commonly sampled sputa because of contamination from upper airway bacteria during sputum expectoration (reviewed in [34]). However, our results show that anaerobes are indeed present within the lower airways, as mucus plugs from one patient's airways contained *Veillonella, Parvimonas*, and *Streptococcus anginosus*, The DNA from these anaerobes was in low abundance, and likely to only be detectable using a method like SI-Seq that offers a great depth of coverage; nevertheless, its presence suggests anaerobic bacteria are within the lower airways of CF patients, and not merely oropharangeal contaminants of sputum. Further analysis will assess the role of these anaerobes in relation to other bacteria present, and the clinical status, antibiotic therapy, and genotype on a larger sample of CF patients.

Although here we describe the use of SI-Seq for characterizing relatively simple CF communities, additional work shows that the approach easily generates sufficient sequence coverage to thoroughly examine more complex communities such as mouse or human feces, house dust, and saliva (unpublished). We believe SI-Seq will be generally applicable to any microbiota. It can be readily adapted by designing primers to target different regions of the 16S rRNA gene, or different loci altogether. This flexibility also opens the possibility of using SI-Seq to study eukaryotic microbes and Archaea.

## Supporting Information

Figure S1 **Flow chart of SI-Seq data analysis pipeline.** Beginning with raw FASTQ reads, the SI-Seq analysis pipeline filters reads based on quality, checks read orientation and corrects orientation if needed, and uses barcode sequences to parse read data into separate output FASTA files.
(TIF)

Figure S2 **Procrustes plot comparison of 454 and SI-Seq community data.** Unweighted UniFrac dissimilarity data were used to generate a Procrustes plot as described in the Materials and Methods, and discussed in the main text and Table 4.
(TIF)

Table S1 **Barcode file.** Barcode sequences designed for SI-Seq development and testing, and for multiplexing CF samples are listed. Barcodes tested with SI-Seq are indicated as such in the ‘Sample’ column.
(XLSX)

Table S2 **Quality filtering and incorrect taxonomic classification.** For the single species controls used in the development of SI-Seq, varying quality cut-off thresholds were used to filter the raw FASTQ data. These results were used to identify the best quality cut-off for maximizing the number of retained reads while minimizing misclassification of reads.
(DOCX)

Table S3 **Example of** ***B. subtilis*** **classifications.** Taxonomy classifications of each read were done using RDP Classifier as discussed in the Materials and Methods. Prior to classification, the reads were filtered based on quality score to exclude any read with more than five sites having a Phred score less than 30 (Table S2).
(DOCX)

## References

[1] Cho I, Blaser MJ (2012) The human microbiome: at the interface of health and disease. Nat Rev Genet 13: 260–270. 22411464 10.1038/nrg3182 PMC3418802

[2] Robinson CJ, Bohannan BJ, Young VB (2010) From structure to function: the ecology of host-associated microbial communities. Microbiol Mol Biol Rev 74: 453–476. 20805407 10.1128/MMBR.00014-10 PMC2937523

[3] Wilson BA, Thomas SM, Ho M (2011) The Human Vaginal Microbiome. In: Nelson KE, editor. Metagenomics of the Human Body. New York: Springer. pp. 91–115.

[4] Bartram AK, Lynch MD, Stearns JC, Moreno-Hagelsieb G, Neufeld JD (2011) Generation of Multimillion-Sequence 16S rRNA Gene Libraries from Complex Microbial Communities by Assembling Paired-End Illumina Reads. Appl Environ Microbiol 77: 3846–3852. 21460107 10.1128/AEM.02772-10 PMC3127616

[5] Caporaso JG, Lauber CL, Walters WA, Berg-Lyons D, Lozupone CA, et al (2011) Global patterns of 16S rRNA diversity at a depth of millions of sequences per sample. Proc Natl Acad Sci U S A 108 Suppl 1 4516–4522. 20534432 10.1073/pnas.1000080107 PMC3063599

[6] Degnan PH, Ochman H (2012) Illumina-based analysis of microbial community diversity. ISME J 6: 183–194. 21677692 10.1038/ismej.2011.74 PMC3246231

[7] Gloor GB, Hummelen R, Macklaim JM, Dickson RJ, Fernandes AD, et al (2010) Microbiome profiling by illumina sequencing of combinatorial sequence-tagged PCR products. PLoS One 5: e15406. 21048977 10.1371/journal.pone.0015406 PMC2964327

[8] Lazarevic V, Whiteson K, Huse S, Hernandez D, Farinelli L, et al (2009) Metagenomic study of the oral microbiota by Illumina high-throughput sequencing. J Microbiol Methods 79: 266–271. 19796657 10.1016/j.mimet.2009.09.012 PMC3568755

[9] Zhou HW, Li DF, Tam NF, Jiang XT, Zhang H, et al (2011) BIPES, a cost-effective high-throughput method for assessing microbial diversity. ISME J 5: 741–749. 20962877 10.1038/ismej.2010.160 PMC3105743

[10] Frank DN (2009) BARCRAWL and BARTAB: software tools for the design and implementation of barcoded primers for highly multiplexed DNA sequencing. BMC Bioinformatics 10: 362. 19874596 10.1186/1471-2105-10-362 PMC2777893

[11] Moustafa A (2010) JAligner: Open source Java implementation of Smith-Waterman. JAligner website. Available: http://jaligner.sourceforge.net. Accessed 2012 June 11.

[12] Dethlefsen L, Huse S, Sogin ML, Relman DA (2008) The pervasive effects of an antibiotic on the human gut microbiota, as revealed by deep 16S rRNA sequencing. PLoS Biol 6: e280. 19018661 10.1371/journal.pbio.0060280 PMC2586385

[13] Sogin ML, Morrison HG, Huber JA, Mark Welch D, Huse SM, et al (2006) Microbial diversity in the deep sea and the underexplored "rare biosphere". Proc Natl Acad Sci U S A 103: 12115–12120. 16880384 10.1073/pnas.0605127103 PMC1524930

[14] Wang Q, Garrity GM, Tiedje JM, Cole JR (2007) Naive Bayesian classifier for rapid assignment of rRNA sequences into the new bacterial taxonomy. Appl Environ Microbiol 73: 5261–5267. 17586664 10.1128/AEM.00062-07 PMC1950982

[15] Edgar RC (2010) Search and clustering orders of magnitude faster than BLAST. Bioinformatics 26: 2460–2461. 20709691 10.1093/bioinformatics/btq461

[16] Caporaso JG, Kuczynski J, Stombaugh J, Bittinger K, Bushman FD, et al (2010) QIIME allows analysis of high-throughput community sequencing data. Nat Methods 7: 335–336. 20383131 10.1038/nmeth.f.303 PMC3156573

[17] Schloss PD, Westcott SL, Ryabin T, Hall JR, Hartmann M, et al (2009) Introducing mothur: open-source, platform-independent, community-supported software for describing and comparing microbial communities. Appl Environ Microbiol 75: 7537–7541. 19801464 10.1128/AEM.01541-09 PMC2786419

[18] Oksanen J, Blanchet FG, Kindt R, Legendre P, Minchin PR, et al.. (2011) vegan: Community Ecology Package.

[19] Glenn TC (2011) Field guide to next-generation DNA sequencers. Mol Ecol Resour 11: 759–769. 21592312 10.1111/j.1755-0998.2011.03024.x

[20] Luo C, Tsementzi D, Kyrpides N, Read T, Konstantinidis KT (2012) Direct comparisons of Illumina vs. Roche 454 sequencing technologies on the same microbial community DNA sample. PLoS One 7: e30087. 22347999 10.1371/journal.pone.0030087 PMC3277595

[21] Minoche AE, Dohm JC, Himmelbauer H (2011) Evaluation of genomic high-throughput sequencing data generated on Illumina HiSeq and Genome Analyzer systems. Genome Biol 12: R112. 22067484 10.1186/gb-2011-12-11-r112 PMC3334598

[22] Armougom F, Bittar F, Stremler N, Rolain JM, Robert C, et al (2009) Microbial diversity in the sputum of a cystic fibrosis patient studied with 16S rDNA pyrosequencing. Eur J Clin Microbiol Infect Dis 28: 1151–1154. 19449045 10.1007/s10096-009-0749-x

[23] Bittar F, Richet H, Dubus JC, Reynaud-Gaubert M, Stremler N, et al (2008) Molecular detection of multiple emerging pathogens in sputa from cystic fibrosis patients. PLoS One 3: e2908. 18682840 10.1371/journal.pone.0002908 PMC2483419

[24] Cox MJ, Allgaier M, Taylor B, Baek MS, Huang YJ, et al (2010) Airway microbiota and pathogen abundance in age-stratified cystic fibrosis patients. PLoS One 5: e11044. 20585638 10.1371/journal.pone.0011044 PMC2890402

[25] Guss AM, Roeselers G, Newton IL, Young CR, Klepac-Ceraj V, et al (2011) Phylogenetic and metabolic diversity of bacteria associated with cystic fibrosis. ISME J 5: 20–29. 20631810 10.1038/ismej.2010.88 PMC3105664

[26] Han MK, Huang YJ, Lipuma JJ, Boushey HA, Boucher RC, et al (2012) Significance of the microbiome in obstructive lung disease. Thorax 67: 456–63. 22318161 10.1136/thoraxjnl-2011-201183 PMC3578398

[27] Harris JK, De Groote MA, Sagel SD, Zemanick ET, Kapsner R, et al (2007) Molecular identification of bacteria in bronchoalveolar lavage fluid from children with cystic fibrosis. Proc Natl Acad Sci U S A 104: 20529–20533. 18077362 10.1073/pnas.0709804104 PMC2154465

[28] Klepac-Ceraj V, Lemon KP, Martin TR, Allgaier M, Kembel SW, et al (2010) Relationship between cystic fibrosis respiratory tract bacterial communities and age, genotype, antibiotics and Pseudomonas aeruginosa. Environ Microbiol 12: 1293–1303. 20192960 10.1111/j.1462-2920.2010.02173.x

[29] Rogers GB, Carroll MP, Serisier DJ, Hockey PM, Jones G, et al (2006) Use of 16S rRNA gene profiling by terminal restriction fragment length polymorphism analysis to compare bacterial communities in sputum and mouthwash samples from patients with cystic fibrosis. J Clin Microbiol 44: 2601–2604. 16825392 10.1128/JCM.02282-05 PMC1489498

[30] Sibley CD, Parkins MD, Rabin HR, Duan K, Norgaard JC, et al (2008) A polymicrobial perspective of pulmonary infections exposes an enigmatic pathogen in cystic fibrosis patients. Proc Natl Acad Sci U S A 105: 15070–15075. 18812504 10.1073/pnas.0804326105 PMC2567494

[31] Sibley CD, Surette MG (2011) The polymicrobial nature of airway infections in cystic fibrosis: Cangene Gold Medal Lecture. Can J Microbiol 57: 69–77. 21326348 10.1139/w10-105

[32] van der Gast CJ, Walker AW, Stressmann FA, Rogers GB, Scott P, et al (2011) Partitioning core and satellite taxa from within cystic fibrosis lung bacterial communities. ISME J 5: 780–791. 21151003 10.1038/ismej.2010.175 PMC3105771

[33] Han MK, Huang YJ, Lipuma JJ, Boushey HA, Boucher RC, et al (2012) Significance of the microbiome in obstructive lung disease. Thorax 67: 456–463. 22318161 10.1136/thoraxjnl-2011-201183 PMC3578398

[34] Lipuma JJ (2010) The changing microbial epidemiology in cystic fibrosis. Clin Microbiol Rev 23: 299–323. 20375354 10.1128/CMR.00068-09 PMC2863368

[35] Huson DH, Auch AF, Qi J, Schuster SC (2007) MEGAN analysis of metagenomic data. Genome Res 17: 377–386. 17255551 10.1101/gr.5969107 PMC1800929

