# Supplementary figures and images for: Analysis of the Cystic Fibrosis Lung Microbiota via Serial Illumina Sequencing of Bacterial 16S rRNA Hypervariable Regions
Source: PLoS One. 2012 Oct 2;7(10):e45791. doi: 10.1371/journal.pone.0045791 (PMC3462755; doi:10.1371/journal.pone.0045791)

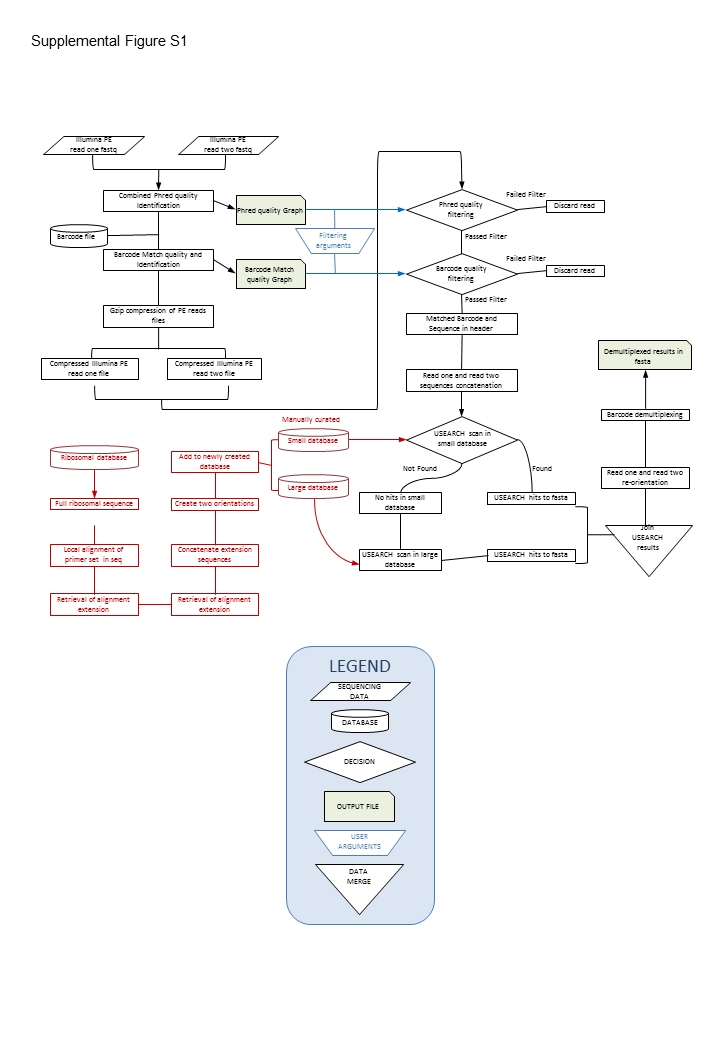

Supplement: Figure S1 — Flow chart of SI-Seq data analysis pipeline. Beginning with raw FASTQ reads, the SI-Seq analysis pipeline filters reads based on quality, checks read orientation and corrects orientation if needed, and uses barcode sequences to parse read data into separate output FASTA files. (TIF) [file pone.0045791.s001.tif]

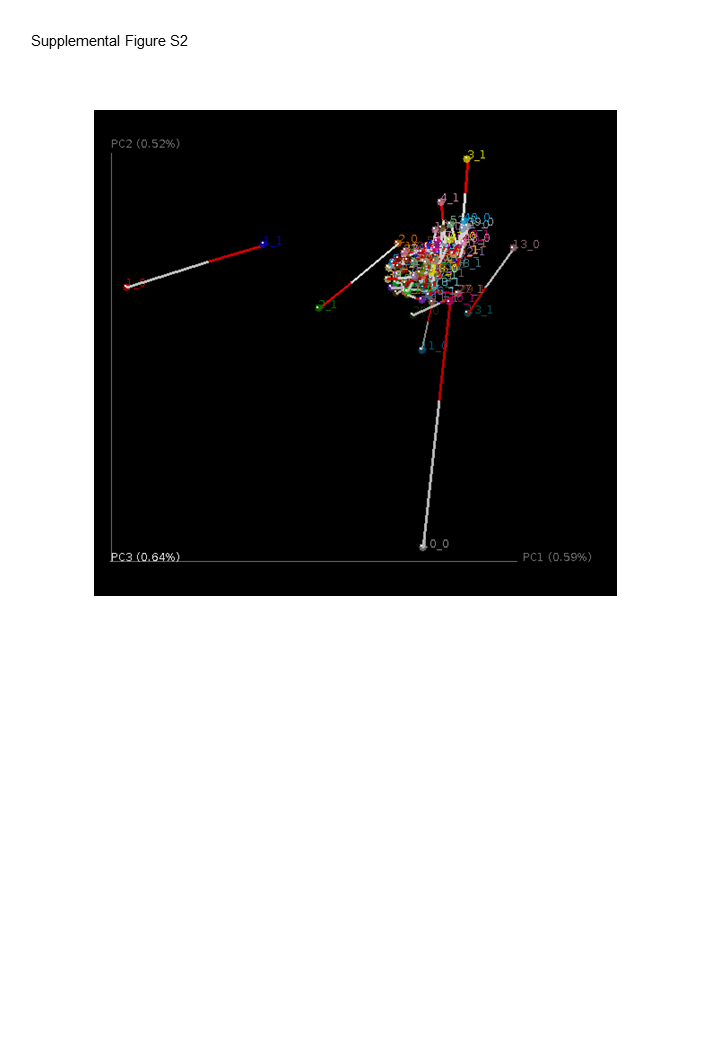

Supplement: Figure S2 — Procrustes plot comparison of 454 and SI-Seq community data. Unweighted UniFrac dissimilarity data were used to generate a Procrustes plot as described in the Materials and Methods, and discussed in the main text and Table 4. (TIF) [file pone.0045791.s002.tif]
