# Supplementary material for: Analysis of the Cystic Fibrosis Lung Microbiota via Serial Illumina Sequencing of Bacterial 16S rRNA Hypervariable Regions
Source: PLoS One. 2012 Oct 2;7(10):e45791. doi: 10.1371/journal.pone.0045791 (PMC3462755; doi:10.1371/journal.pone.0045791)
Supplement: Table S2 — Quality filtering and incorrect taxonomic classification. For the single species controls used in the development of SI-Seq, varying quality cut-off thresholds were used to filter the raw FASTQ data. These results were used to identify the best quality cut-off for maximizing the number of retained reads while minimizing misclassification of reads. (DOCX) [file pone.0045791.s004.docx]

| Supporting Table S2. Quality filtering and incorrect taxonomic classification. | | | | | |
| --- | --- | --- | --- | --- | --- |
| Species | Total Reads | Quality cutoff | Quality reads | Percent Quality | Percent reads misclassified |
| *P. aeruginosa* | 17,520,529 | 5 sites < 30 | 13,530,371 | 77% | 0.08% |
| *P. aeruginosa* | 17,520,529 | 2 sites < 30 | 9,281,166 | 53% | 0.06% |
| *P. aeruginosa* | 17,520,529 | 2 sites < 33 | 983,819 | 6% | 0.02% |
| *P. aeruginosa* | 17,520,529 | 2 sites < 35 | 45,709 | 0.3% | 0.006% |
| *B. subtilis** | 13,524,480 | 5 sites < 30 | 9,285,467 | 69% | 0.03% |
| *B. subtilis* | 13,524,480 | 2 sites < 30 | 3,947,584 | 29% | 0.02% |
| *B. subtilis* | 13,524,480 | 2 sites < 33 | 994,002 | 7% | 0.02% |
| *B. subtilis* | 13,524,480 | 2 sites < 35 | 93,831 | 0.7% | 0.02% |
| *Taxonomy classifications shown in Supporting Table S3. | | | | | |
