# Supplementary material for: Analysis of the Cystic Fibrosis Lung Microbiota via Serial Illumina Sequencing of Bacterial 16S rRNA Hypervariable Regions
Source: PLoS One. 2012 Oct 2;7(10):e45791. doi: 10.1371/journal.pone.0045791 (PMC3462755; doi:10.1371/journal.pone.0045791)
Supplement: Table S3 — Example of B. subtilis classifications. Taxonomy classifications of each read were done using RDP Classifier as discussed in the Materials and Methods. Prior to classification, the reads were filtered based on quality score to exclude any read with more than five sites having a Phred score less than 30 (Table S2). (DOCX) [file pone.0045791.s005.docx]

| Supporting Table S3. Example of *B. subtilis* classifications shown for the dataset marked with * in Supporting Table S2. | | |
| --- | --- | --- |
| Taxonomy | Number of  Reads Assigned | Proportion of  Total Reads |
| *Bacillus* | 7,991,160 | 0.86060938 |
| *Bacillaceae* | 1,290,946 | 0.139028656 |
| *Bacteria* | 847 | 9.12178E-05 |
| *Clostridiales* | 515 | 5.5463E-05 |
| *Firmicutes* | 505 | 5.43861E-05 |
| *Lachnospiraceae* | 214 | 2.30468E-05 |
| *Bacteroidetes* | 203 | 2.18621E-05 |
| *Pseudomonas* | 176 | 1.89544E-05 |
| *Lactobacillus* | 124 | 1.33542E-05 |
| *Ruminococcaceae* | 109 | 1.17388E-05 |
| *Helicobacter* | 95 | 1.0231E-05 |
| *Oscillibacter* | 89 | 9.58487E-06 |
| *Staphylococcus* | 52 | 5.60015E-06 |
| *Parasutterella* | 36 | 3.87703E-06 |
| *Clostridia* | 35 | 3.76933E-06 |
| *Bacteroides* | 31 | 3.33855E-06 |
| *Proteobacteria* | 30 | 3.23086E-06 |
| *Alistipes* | 23 | 2.47699E-06 |
| *Enterobacteriaceae* | 20 | 2.1539E-06 |
| *Bacillales* | 20 | 2.1539E-06 |
| *Lactobacillales* | 19 | 2.04621E-06 |
| *Bacilli* | 18 | 1.93851E-06 |
| *Burkholderia* | 17 | 1.83082E-06 |
| *Barnesiella* | 16 | 1.72312E-06 |
| *Robinsoniella* | 15 | 1.61543E-06 |
| *Gammaproteobacteria* | 14 | 1.50773E-06 |
| *Prevotella* | 13 | 1.40004E-06 |
| *Erwinia* | 13 | 1.40004E-06 |
| *Butyricicoccus* | 13 | 1.40004E-06 |
| *Paraliobacillus* | 10 | 1.07695E-06 |
| *Coriobacteriaceae* | 8 | 8.61561E-07 |
| *Bifidobacterium* | 7 | 7.53866E-07 |
| *Bacteroidales* | 7 | 7.53866E-07 |
| *Porphyromonadaceae* | 6 | 6.46171E-07 |
| *Deltaproteobacteria* | 6 | 6.46171E-07 |
| *Sporobacterium* | 4 | 4.30781E-07 |
| *Turicibacter* | 3 | 3.23086E-07 |
| *Prevotellaceae* | 3 | 3.23086E-07 |
| *Parabacteroides* | 3 | 3.23086E-07 |
| *Gordonibacter* | 3 | 3.23086E-07 |
| *Burkholderiales* | 3 | 3.23086E-07 |
| *Blautia* | 3 | 3.23086E-07 |
| *Veillonella* | 2 | 2.1539E-07 |
| *Streptomycetaceae* | 2 | 2.1539E-07 |
| *Pseudomonadaceae* | 2 | 2.1539E-07 |
| *Oribacterium* | 2 | 2.1539E-07 |
| *Neisseria* | 2 | 2.1539E-07 |
| *Haemophilus* | 2 | 2.1539E-07 |
| *Erysipelotrichaceae* | 2 | 2.1539E-07 |
| *Enterorhabdus* | 2 | 2.1539E-07 |
| *Dorea* | 2 | 2.1539E-07 |
| *Campylobacter* | 2 | 2.1539E-07 |
| *Butyricimonas* | 2 | 2.1539E-07 |
| *Virgibacillus* | 1 | 1.07695E-07 |
| *Sporobacter* | 1 | 1.07695E-07 |
| *Porphyromonas* | 1 | 1.07695E-07 |
| *Mucispirillum* | 1 | 1.07695E-07 |
| *Megasphaera* | 1 | 1.07695E-07 |
| *Granulicatella* | 1 | 1.07695E-07 |
| *Gemella* | 1 | 1.07695E-07 |
| *Fusobacterium* | 1 | 1.07695E-07 |
| *Escherichia/Shigella* | 1 | 1.07695E-07 |
| *Cedecea* | 1 | 1.07695E-07 |
| *Actinomyces* | 1 | 1.07695E-07 |
